# Whole Exome Sequencing of Intracranial Epidermoid Cysts Reveals Immune-Associated Mechanistic and Potential Targets

**DOI:** 10.3390/cancers16203487

**Published:** 2024-10-15

**Authors:** Shruthi Kondaboina, Oscar Parrish, Carolina Angelica Parada, Manuel Ferreira

**Affiliations:** Department of Neurological Surgery, University of Washington Medical Center 1, Seattle, WA 98195, USA; skonda2@uw.edu (S.K.); orp@uw.edu (O.P.)

**Keywords:** intracranial epidermoid cysts, rare tumors, whole exome sequencing, targeted therapy

## Abstract

**Simple Summary:**

Intracranial Epidermoid Cysts (IECs) are rare tumors. Despite their benign course, IECs can adhere to critical brain structures resulting in impairment and morbidity. The primary treatment is surgery; however, cyst adherence often complicates complete removal, resulting in notably high progression rates after subtotal resection. Due to the rarity of IECs, there has been limited research focused on understanding the mechanisms of the disease and advancing therapeutic options. As a result, there are currently no effective drug therapies available for treating patients with IECs. Targeted therapy is an effective form of treatment for tumors. It focuses on mutations that turn healthy cells into tumor cells. The mutation profile of IECs has not been investigated. We aimed to investigate the somatic landscape of IECs to gain insights into tumor biology and identify mutations that could potentially serve as targets for additional therapies. Surgery is still the most effective treatment for these patients.

**Abstract:**

**Background/Objectives:** Intracranial Epidermoid Cysts (IECs) are rare intracranial tumors primarily treated through surgery. Cyst adherence complicates complete removal, leading to high rates of tumor progression after subtotal resection. The molecular drivers of IEC remain unknown. Consequently, advances in treatment have fallen short. Tumor genetic profiling has revealed potential targets for drug development, including FDA-approved options and reshaping treatment. The genetic landscape of IECs has not been explored. We applied Whole Exome Sequencing (WES) to IECs to gain insights into the mechanisms of oncogenesis and identify potential therapeutic targets. **Methods:** We performed WES on tumor tissue and matched blood samples, when available. Following GATK best practices, we conducted read processing, quality control, somatic variant calling, and copy-number inference. Data analyses and visualization were conducted in R. **Results:** Top altered genes are associated with the immune system and tumor microenvironment, suggesting a mechanism of immune evasion. Gene and pathway enrichment revealed a high mutation burden in genes associated with Extracellular Matrix (ECM) and PI3K-AKT-mTOR cascades. Recurrent and deleterious alterations in NOTCH2 and USP8 were identified in 50% and 30% of the cohort, respectively. Frequent amplifications in deubiquitinases and beta-defensins strengthened the involvement of immune mechanisms for oncogenic transformation. **Conclusions:** Top altered genes and recurrent mutations may play a role in shaping the microenvironment and modulating immune evasion in IECs. USP8 and NOTCH2 may serve as clinically relevant target for IECs. Finally, we present evidence that the crosstalk between the PI3K-Akt-mTOR and ECM signaling pathways may play a role in modulating the immune escape mechanism in IECs.

## 1. Introduction

Intracranial Epidermoid Cysts (IECs) are congenital ectodermal inclusions that arise from incomplete closure of the neural tube during fetal brain development [1,2]. IECs are rare brain tumors representing only 0.3–1.8% of all intracranial tumors [2,3]. They typically occur in both genders, between the ages of 20–60 years, with a peak incidence in the fourth decade [4]. In the skull base, IECs are often located in the cerebellopontine angle (37.3%) and parasellar region (30%), spreading in the subarachnoid space of the basal cisterns [5]. They may also be found in the middle cranial fossa (18%), diploe (16%), spinal canal (5%) [5,6], and rarely in the brain stem [7,8,9]. Magnetic resonance imaging (MRI) is the diagnostic approach to identify IECs. Diffusion-weighted MRI allows greater accuracy in the preoperative differential diagnosis [10]. 

IECs are usually slow-growing and indolent tumors. Despite their benign nature, they can undergo malignant transformation. Tumor growth can be driven by the division of the stratified squamous epithelium lining its cavity. Cyst contents are largely composed of acellular keratin debris and cholesterol inclusions. Liquefaction of the cyst contents is associated with infection or loss of vascularity [11]. Aggressive IECs have a propensity to adhere to critical neurovascular structures, resulting in significant morbidity and neurologic deficit [12,13]. Malignant transformation of IECs into secondary malignant lesions has been reported [14,15,16], including the development of melanoma [17] and Squamous Cell Carcinoma (SCC) [18]. 

Surgery becomes necessary when IECs exhibit characteristics of malignant transformation, such as rapid growth, adherence to or invasion of critical brain structures, recurrence/progression, and, in rare cases, metastasis. Gross total resection is critical to minimize tumor progression [19]; however, cyst adherence results in difficult removal. A subtotal resection yields progression rates of up to 93% [20]. Radiation does not appear to play a significant role in the management of IECs; however, it has been used as an adjunct therapy for cysts in an effort to control potential malignant transformation. Due to the rarity of IECs, research focusing on cyst prognosis, diagnosis, and treatment is limited. Challenges include a lack of timely access to molecular testing for determining eligibility for targeted therapies, difficulties in enrolling sufficient numbers of patients in clinical trials, and limited incentives for drug development. As a result of the insufficient understanding of the pathophysiology and molecular characteristics of IECs, effective systemic or targeted therapies for these patients are not currently available.

Next-generation sequencing has dramatically reshaped oncological treatment through the identification of genetic variants that provide prognostic information, and aid in therapeutic selection, resulting in hypothesis-driven clinical trials [21]. Many genetic variants may be targeted by FDA-approved options, highlighting the potential repositioning of these drugs to a new indication [22].

The genetics of IECs remain underexplored. There have been suggestions of a familial inheritance pattern observed for epidermoid cysts of the spleen [23]. Mice lacking the IL-1 receptor (IL-1R) (IL1r^−/−^) or deficient in IL1-β developed immunosuppression and multiple epidermal cysts after chronic UVB [24], suggesting that induced somatic events and an altered innate immune response may be involved in the initiation of epidermoid cysts. However, the role of somatic genetic variants in the development and progression of skull base epidermoid cysts is unknown.

In this study, we applied Whole Exome Sequencing (WES) to investigate somatic alterations in IECs that required surgical intervention due to their aggressive features. Our primary goal was to improve our understanding of the pathophysiology underlying their malignant transformation. Gaining insight into the mechanisms of cyst development and progression could lead to the identification of potentially actionable variants with clinical relevance, ultimately advancing treatment options for patients who currently rely on surgery as the most effective intervention.

## 2. Materials and Methods

### 2.1. Specimens, Patients, and Clinical Data

Studies were conducted following the U.S. Common Rule ethical guidelines. Tumor was resected from participants with the diagnosis of IEC who underwent surgery between 1995 and 2021 at the University of Washington hospitals (Seattle, WA, USA). The respective clinical data was extracted from the University of Washington, School of Medicine clinical database. Data and specimen collection were reviewed and approved by the University of Washington Institutional Review (STUDY 00002162). Written informed consent was obtained from all subjects. Samples were collected and stored in tumor biobank until further processing. Specimens were reviewed by three neuropathologists and neurosurgeons. The pathology reports confirmed the diagnosis of IECs with aggressive features. Clinical data was gathered regarding date, history, demographics, imaging, neuropathology reports, operative information, pre-and post-operative symptoms, adjuvant treatment, and outcomes (Supplementary Table S1). Recurrence was defined as at least 1 cm of enhancement on MRI subsequent gross total resection. Progression was considered to be, at least, 1 cm of growth of a residual tumor on MRI after partial surgical resection. 

### 2.2. Genomic DNA Storage and Handling

Genomic DNA (gDNA) was isolated from six fresh-frozen tissues and matching blood (when available) using the QIAmp DNA Mini Kit (Qiagen, Hilden, Germany) and QIAmp DNA Blood Mini Kit (Qiagen, Hilden, Germany), respectively, following the manufacturer’s recommended protocol. gDNA samples were isolated post-surgically. Proper storage and handling are essential to the maintenance of quality DNA for downstream research applications. gDNA was stored in aliquots to avoid freeze-thawing. Samples were stored at a temperature below −80 °C to maintain integrity during long-term storage. 

### 2.3. Whole-Exome Sequencing 

DNA Quality Control (QC), library preparation and sequencing of gDNA was carried out at the University of Washington Northwest Genomics Center, Seattle, WA. DNA was quantified using the Qubit fluorometer (Invitrogen, Carlsbad, CA, USA). DNA quality control included DNA quantification, values of absorbance ratio A_260_/A_280_, sex typing, and molecular “fingerprinting” using TaqMan Open Array 63-SNP assay (Thermo Fischer Scientific, Waltham, MA, USA) using a custom exome SNP (Northwest Genomics). Samples fail QC if: (1) the total amount, concentration, or volume is too low; (2) the fingerprint assay produces poor genotype data or integrity of DNA; or (3) sex-typing is inconsistent with the sample. All samples passed QC. Automated library construction and exome capture were carried out in 96-well plate format, using Perkin-Elmer Janus II (PerkinElmer, Waltham, MA, USA) equipment. Five hundred nanograms of genomic DNA were subjected to a series of shotgun library construction steps, including fragmentation through acoustic sonication (Covaris, Woburn, MA, USA), end-polishing, and A-tailing ligation of sequencing adaptors, followed by PCR amplification with dual 10 bp barcodes for multiplexing. Libraries underwent exome capture using the Twist Human Core Exome Kit + RefSeq exome (36.5 MB target) (Twist Biosciences, San Francisco, CA, USA). Briefly, 187.5 ng of the shotgun library was hybridized to biotinylated capture probes for 16–18 h. Enriched fragments were recovered via streptavidin beads and PCR was amplified. Since each library was uniquely barcoded, samples were captured in multiplex. Before sequencing, the pooled library concentration was determined by fluorometric assay. Molecular weight distributions were verified on the Agilent Bioanalyzer (Agilent, Santa Clara, CA, USA) (consistently 180 ± 15 bp). Parallel sequencing-by-synthesis with fluorescently labeled, reversibly terminating nucleotides was carried out on the NovaSeq6000 instrument (RTA 3.1.5) (Illumina, San Diego, CA, USA) in Paired-End 100 bp (PE100) mode, using NovaSeq 6000 v1.5 Reagent Kit (Illumina, San Diego, CA, USA).

### 2.4. Read Processing, Quality Control, and Somatic Variant Calling

Base calls generated in real-time on the NovaSeq6000 were demultiplexed. Raw reads were assessed for Phred score quality using the FastQC tool kit v0.11.9 (https://www.bioinformatics.babraham.ac.uk/projects/fastqc/, accessed on 21 September 2023). Trimmomatic v0.3.9 [25] was used to detect and remove sequencing adapters, primers, and low-quality nucleotides. Trimmed reads were aligned against the reference human genome build hg38 (UCSC Genome Browser) [26] using the Burrows-Wheeler Aligner (BWA) (v0.7.15) [27]. All aligned read data were sorted and subject to duplicate removal using Picard (v2.6.0) (https://broadinstitute.github.io/picard/, accessed on 21 September 2023). Base qualities were recalibrated with GATK BaseRecalibrator (v4.2.6.1) (https://gatk.broadinstitute.org/, accessed on 21 September 2023). Somatic variant calling in unmatched samples was performed against the GATK panel of normal (PoN) (human-built hg38) using three variant callers: Mutect2 (v4.2.6.1) (GATK), Varscan (v2.4.2) [28], and Vardict v2019.06.04-0 [29]. Tumor/normal variant calling was conducted with Mutect2 (v4.2.6.1) (GATK), Varscan (v2.4.2) [28], MuSe (v1.0), and Strelka2 v2.9 [30]. To identify high-confidence mutations, a joint analysis was applied by combining at least three software (three-caller pipeline approach) [31]. Mutation calls were selected through a stringent filtering process and functionally annotated with ANNOVAR v 2020-06-08 [32]. See also Supplementary Table S2.

### 2.5. Somatic Copy-Number Inference

Somatic Copy Number Variations (CNVs) were identified using CNVkit (v0.9.9) [33]. Whole-exome sequencing read alignments in BAM format, the capture bait locations, and the reference human genome build hg38 (UCSC Genome Browser) [26] were the inputs to the program. All additional files used in the copy-number workflow, such as GC content and the location of sequence repeats, were extracted from the genome sequence in FASTA format using scripts included with the CNVkit v0.9.9 distribution. Log_2_ copy ratios across the genome for each sample were calculated based on on-target reads and the nonspecifically captured off-target reads. The baseline of normalized sequencing depth on targeted regions was first constructed based on normal samples. For each tumor sample, log2 copy number ratio of normalized sequencing depth on each targeted region between the tumor sample and the baseline was calculated. A CNVKit built-in segmentation algorithm was applied to the log_2_ ratio values to infer discrete copy number segments [33]. The log_2_ ratios and segments were used as inputs for the genomic Identification of Significant Targets in Cancer (GISTIC) v2.0.23 [34], allowing the identification of copy number regions that were significantly amplified or deleted across the set of samples. 

### 2.6. Data Analysis and Visualization

Genomic data analysis, visualization, and annotation were conducted in R v4.1.2. Gene Ontology (GO) was performed by using Bioconductor v3.1.7, clusterProfiler v4.2.2 [35] and enrichR browser (https://maayanlab.cloud/Enrichr/, accessed on 16 December 2023) [36] with the inclusion of Molecular Function, Biological Process, and Molecular Component databases [37]. The parameters for GO analysis included the list of all 728 altered genes identified in IECs, a q-value cutoff of 0.1, and a Benjamini and Hochberg (BH) adjusted *p*-value cutoff of 0.05. Pathway enrichment analysis was carried out using the Kyoto Encyclopedia of Genes and Genomes (KEGG) 2021 Human database [38]. Reactome 2022 [39] and WikiPathways 2024 [40] databases were also assessed. Search Tool for the Retrieval of Interacting Genes (STRING-DB V12.0, http://www.string-db.org/, accessed on 11 October 2024) was used to construct the Protein-Protein Interaction (PPI) network using full STRING network, medium confidence, FDR stringency of 5% and k-means clusterization. We utilized the Maftools v2.10.05 [41] package to extract, analyze, and visualize copy-number aberrations and mutational signatures. *p* < 0.05 was considered statistically significant unless stated otherwise. Biorender software v4 (Toronto, CA, USA) was used for scientific illustration. See also Supplementary Table S2.

All software versions, source code, and databases used in the present study are listed in Supplementary Table S2.

Data will be deposited in a publicly available database before publication.

## 3. Results

### 3.1. Samples and Clinical Data

We performed WES in six IEC tissue samples. The six samples used in this study were collected from patients who underwent surgical resection of an IEC from 1995 to 2021 at the University of Washington hospitals (Table 1 and Supplementary Table S1). WES was also performed in the gDNA of matching blood samples, if available. The mean age of the patient cohort was 36.8 years, with all but one individual being of female sex. Fifty per cent of the patients presented with symptoms of headaches, while 33% experienced changes in vision before surgical intervention. After diagnosis, all patients underwent craniotomy for tumor resection. Among them, 50% of the cases underwent Gross Total Resection (GTR), two patients (33%) received Subtotal Resection (STR), and one individual (17%) underwent a near Gross Total Resection (nGTR) (Table 1). In H766, an STR posed a progression risk, diagnosed ~7 months post-STR. For 4316, two Gross Total Resections (GTR) in 2011, and 2016 resulted in tumor recurrence. The complete clinical information is presented in the Supplementary Table S1.

### 3.2. Overview of the Somatic Mutational Signature of IECs

**Figure 1:**
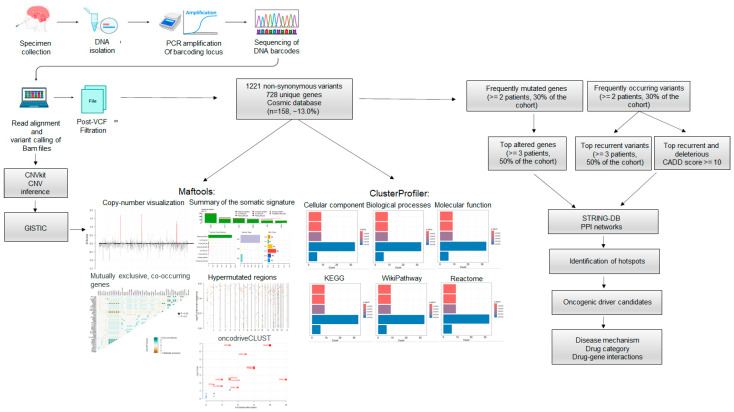
Whole Exome Sequencing (WES) and data analysis workflow. WES was conducted on six fresh-frozen epidermoid cysts. The sequencing reads were aligned to the reference human genome hg38. Aligned BAM files underwent copy number inference using CNVkit and somatic variant calling. A total of 1221 alterations across 728 unique genes were identified, with nearly 13.0% (*n* = 158/1221) of these mutations reported in COSMIC. Gene Ontology (GO) annotation and pathway enrichment were performed on the 728 altered genes. The analysis of frequently mutated genes and common variants enabled the identification of protein-protein interaction (PPI) networks, mutation hotspots, and potential oncogenic drivers. Disease mechanisms and possible targeted therapeutic options were suggested.

The WES workflow and data analysis are shown in Figure 1. We performed the gDNA isolation of six fresh-frozen epidermoid samples resected during surgery and respective matching blood, when available. After library preparation, the WES was carried out. Read alignment, somatic variant calling, and VCF filtration were carried out as described in the methods. For translational purposes, only non-synonymous alterations and indels were considered for downstream analysis. A total of 1221 variants corresponding to 1094 unique alterations and affecting 728 unique genes were identified (Supplementary Table S3). From these, ~13.00% (158/1221) have been reported in the Catalogue of Somatic Mutations in Cancer—COSMIC database (https://cancer.sanger.ac.uk/cosmic, accessed on 21 September 2023) (Figure 1).

**Figure 2:**
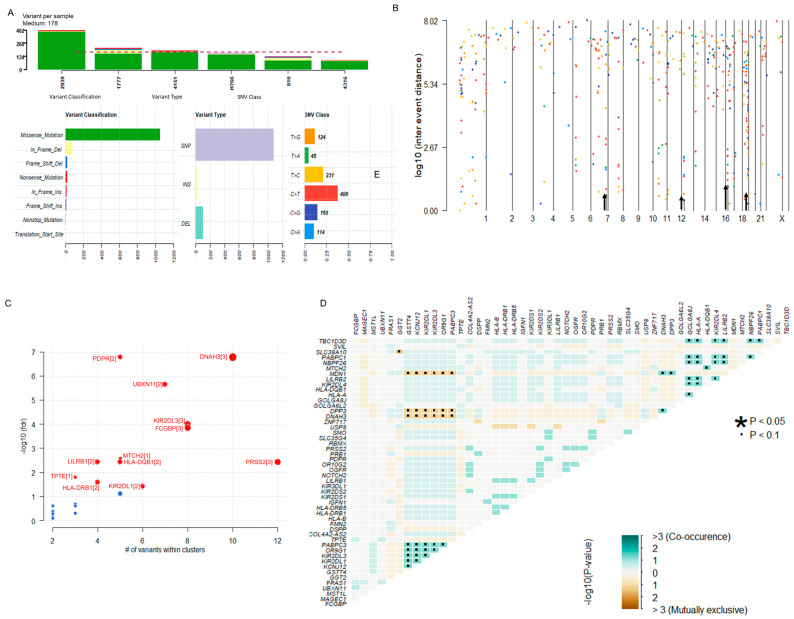
Overview of the Somatic Mutational Signature of IECs. (**A**) Data Summary: The bar plots summarize the number of variants identified in each sample, including the variant classification, variant type, and SNV class in the dataset. The median number of variants per sample was 178. Most variants identified were missense mutations (1065/1221, 87.22%) and SNPs (1092/1221, 89.43%). Predominant SNV alterations included C > T (*n* = 408) and T > C (*n* = 231). (**B**) Hypermutated regions. The rainfall plot of log10(inter-event distance) and chromosome number show regions where potential changes in inter-event distances are located (also indicated by an arrow). Each point of the rainfall plot is color-coded according to the SNV class. Changes in inter-event distances (arrow) are located on chromosomes 7, 12, 16, and 19. (**C**) Prediction of Disease-Associated Driver Genes. The bubble plot of -log10(fdr) and the number of variants within clusters show the predicted driver candidates (marked in red). The number of closely spaced mutational clusters is highlighted within brackets. (**D**) Mutually Exclusive or Co-occurring genes: the triangular matrix displays relationships among the top mutated genes (*p* < 0.1). Green indicates a tendency toward co-occurrence, whereas pink indicates a tendency toward exclusiveness. See also Supplementary Table S3.

The summary of the somatic mutational signature of IECs is shown in Figure 2. The median number of variants per sample was 178. The majority of variants identified were missense mutations (1065/1221, 87.22%). In-frame deletions and insertions corresponded to 72/1221 (6.47%) and 15/1221 (1.23%), respectively. Frame shift mutations constituted 2.7% (33/1221) of all somatic alterations. Nonsense and nonstop mutations corresponded to 22/1221 (1.80%) and 5/1221 (0.41%), respectively. Two (0.16%) translation start site alterations were also identified. According to the variant type, 89.43% (1092/1221) were Single Nucleotide Polymorphisms (SNPs), 2.21% (27/1221) were insertions, and 8.35% (102/1221) were deletions. The most predominant Single Nucleotide Variant (SNV) class alterations were C > T (*n* = 408) and T > C (*n* = 231) (Figure 2A). Hypermutated regions were explored by plotting inter-variant distances on a linear genomic scale. Visualization through a rainfall plot revealed changes in inter-event distances located on chromosomes 7, 12, 16, and 19 (Figure 2B). Potential driver candidates identified by the algorithm oncodriveCLUST [42] included the genes PDPR, DNAH2, UBXN11, KIR2DL1/3, FCGBP, PRSS2, MTCH2, LILRB1, HLA-DQB1, HLA-DRB1, and TPTE (Figure 2C). Pair-wise Fisher’s Exact of the top fifty altered genes of the dataset detected pairs of mutually exclusive or co-occurring sets of mutated genes. Relationships were identified when *p* < 0.1 (Figure 2D).

### 3.3. IECs Are Characterized by an Altered Immune Repertoire

**Figure 3:**
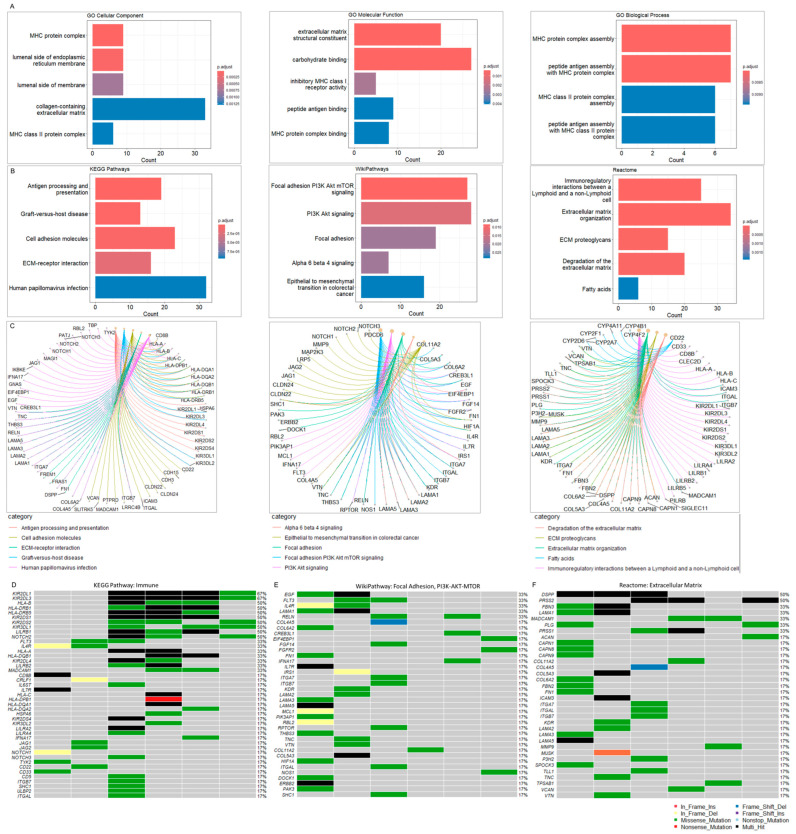
(**A**) Gene Ontology (GO) analysis. GO was performed with the inclusion of Molecular Function, Biological Process, and Molecular Component databases. The bar plots show the top enriched terms. Bars correspond to terms with significant adjusted *p*-values < 0.05. (**B**) Pathway enrichment analysis. The bar plots show the top enriched terms in each library (KEGG 2021, WikiPathway 2024, and Reactome 2022). Colored bars correspond to terms with significant adjusted *p*-values < 0.05. (**C**) Pathway gene networks. Pathway categories are color-coded to facilitate the visualization of the genes associated with each significant term. (**D**) Oncoplot of altered immune-associated genes. The oncoplot illustrates the most altered immune-associated genes within the cohort. (**E**) Oncoplot of altered focal adhesion/PI3K-Akt-mTOR-associated genes. (**F**) Oncoplot of Extracellular Matrix (ECM)-associated genes. The oncoplots illustrate the most altered immune-associated genes within the cohort. The oncoplot illustrates the most altered immune-associated genes within the cohort. The legend shows color-coded variant classification. Variants labeled as Multi_Hit indicate genes that are mutated more than once within the same sample. See also Supplementary Tables S4–S7.

GO annotation and pathway analyses were performed on the 728 altered genes. The top five cellular components involved the MHC protein complex, the luminal side of the endoplasmic reticulum membrane, the collagen-containing extracellular matrix, and the MHC class II protein complex. The molecular function annotation showed extracellular matrix structural constituent, carbohydrate binding, inhibitory MHC class I receptor activity, peptide binding antigen, and MHC protein complex binding. The main biology processes were associated with the MHC protein complex assembly, peptide antigen assembly with the MHC protein complex, MHC class II protein complex assembly, and peptide antigen assembly with the MHC class II protein complex (Figure 3A) (Supplementary Table S4). Pathway enrichment analysis was carried out using KEGG [38], Reactome [39], and WikiPathways [40] (Figure 3B). The top five KEGG pathways included antigen processing and presentation, graft-versus-host disease, cell adhesion molecules, Extracellular Matrix (ECM)-receptor interaction, and human papillomavirus infection. The KEGG pathway enrichment and respective associated genes are presented in Figure 3C and Supplementary Table S5. 

The top WikiPathways involved focal adhesion PI3K-Akt-mTOR, PI3K-Akt-mTOR, and focal adhesion pathways, followed by the alpha-6-beta-4 and epithelial to mesenchymal transition in colorectal cancer cascade. The WikiPathways enrichment and associated genes are presented in Figure 3C and Supplementary Table S6. The analysis with Reactome revealed immunoregulatory interactions between lymphoid and non-lymphoid cells, ECM organization, ECM proteoglycans, degradation of the ECM and fatty acids signaling. The Reactome enrichment and associated genes are presented in Figure 3C and Supplementary Table S7.

Both GO and pathways enrichments demonstrated that many altered genes in IECs are closely associated with immunity and ECM, suggesting the involvement of the immune system and microenvironment as a mechanism of intracranial epidermoid cyst formation. 

To better visualize alterations in the immune repertoire, we assessed genetic changes in immune-related genes using an oncoplot (Figure 3D). Remarkably, somatic mutations in these genes were present in 100% of the cohort. The most frequently altered immune-related genes in IECs were Killer cell immunoglobulin-like receptors 2DL1 (KIR2DL1) and 2DL3 (KIR2DL3), which harbored multiple missense and multi-hit mutations, affecting approximately 70% of IEC patients. Additionally, we observed DNA variations in other Killer cell immunoglobulin-like receptors (KIR2DS1/2/4, KIR3DL1/2, KIR2DL4), HLA genes (HLA-A/B/C, HLA-DRB1/5, HLA-DQB1, HLA-DPB1, HLA-DQA1/2), and other immune-associated genes, albeit at lower frequencies within the study cohort (Figure 3D, Supplementary Table S3). These alterations in the immune repertoire associated with IECs strongly suggest mechanisms of tumor immune evasion. 

IECs display a high mutation burden in genes associated with ECM-mediated cellular interactions and PI3K-AKT-mTOR signaling.

Next, we assessed the mutation burden in genes associated with PI3K-Akt-mTOR signaling and ECM-mediated cellular interactions as enriched by WikiPathways and Reactome. Somatic mutations on these genes also affected 100% of the cohort.

The top altered genes associated with the focal Adhesion and PI3K-Akt-mTOR signaling included the Epidermal Growth Factor (EGF), Receptor-type tyrosine-protein kinase FLT3 (FLT3), Interleukin-4 receptor subunit alpha (IL4R), Laminin subunit alpha-1 (LAMA1), and Reelin (RELN) (~33%) (Figure 3E, Supplementary Table S3). The top altered genes related to the extracellular matrix included the Dentin sialophosphoprotein (DSPP) and Trypsin-2 (PRSS2) (Figure 3F, Supplementary Table S3). 

ECM components and the PI3K-Akt-mTOR signaling pathway play crucial roles in processes related to tumor progression, including cell proliferation, adhesion, invasion, and migration [43,44]. Aberrant interactions between EGF and ECM receptors lead to the downstream activation of the PI3K-Akt-mTOR pathway [43]. The high mutation burden observed in genes strongly suggests a potential interplay between PI3K-Akt-mTOR signaling and ECM, contributing to tumor cell function and the establishment of a tumor-supportive microenvironment in IECs.

Gene Ontology (GO) was performed by using Bioconductor v3.1.7, clusterProfiler v4.2.2 [35] and enrichR browser (https://maayanlab.cloud/Enrichr/, accessed on 11 October 2024) [36] with the inclusion of Molecular Function, Biological Process, and Molecular Component databases [37]. The parameters for GO analysis included the list of all 728 altered genes identified in IECs, a q-value cutoff of 0.1, and a Benjamini and Hochberg (BH) adjusted *p*-value cutoff of 0.05. Pathway enrichment analysis was carried out using the Kyoto Encyclopedia of Genes and Genomes (KEGG) 2021 Human database [38]. Reactome 2022 [39] and WikiPathways 2024 [40] databases were also assessed.

### 3.4. Oncogenic Driver Candidates of IECs

**Figure 4:**
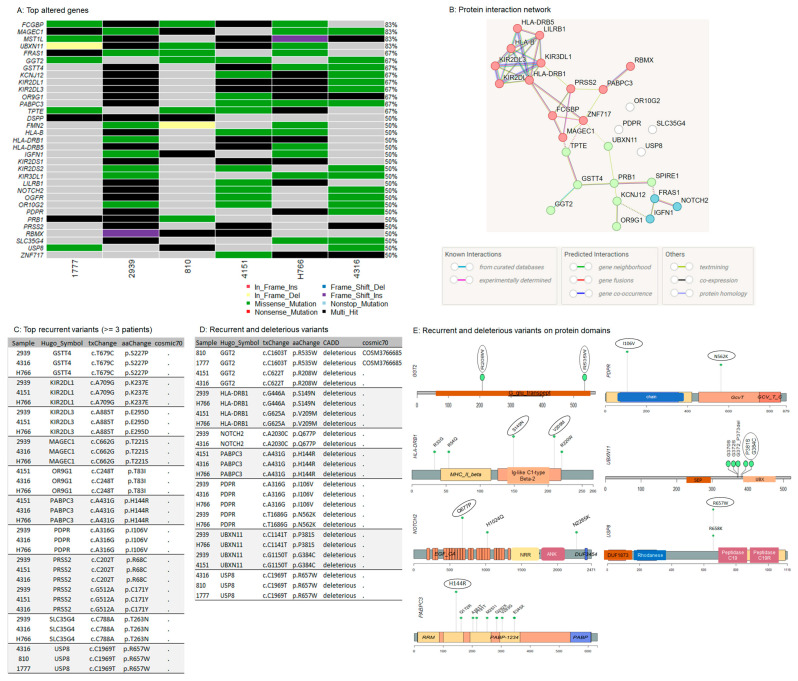
Potential oncogenic drivers and recurrent somatic variants in IECs. (**A**) Top altered genes in IECs. The oncoplot illustrates the most altered genes within the cohort. The legend shows color-coded variant classification. Variants labeled as Multi_Hit indicate genes that are mutated more than once within the same sample. (**B**) Interaction network. The protein-protein interaction (PPI) network illustrates the functional associations among the top altered genes. This PPI analysis was conducted using the STRING tool, applying a medium confidence threshold, an FDR stringency of 5%, and k-means clustering. (**C**) Top recurrent variants: list of the most recurrent somatic events, affecting at least 50% (*n* = 3/6) of the cohort. (**D**) Recurrent and predicted deleterious variants: list of recurrent variants predicted as deleterious according to the Combined Annotation Dependent Depletion (CADD). (**E**) Lollipop plots of recurrent and predicted deleterious variants on protein domains. The circle indicates the most frequently occurring mutations within the cohort. See also Supplementary Tables S3 and S8.

To identify highly mutated genes that are likely oncogenic drivers of IECs, we focused on genes altered in at least 50% of the cohort. (≥3 patients). Thirty-three genes met the criteria (Figure 4A, Supplementary Table S8). Except for Basic Salivary Proline-Rich Protein 1 (PRB1), which is not associated with cancer hallmarks according to the literature, all other identified genes have been previously linked to oncogenic transformation. Furthermore, over 50% of these genes have been previously linked to tumor immune infiltration and the tumor microenvironment (Table 2), reinforcing their potential role as oncogenic drivers. The malignant transformation of IECs into Squamous Cell Carcinoma (SCC) and melanoma has been previously reported [17,18]. Notably, approximately 36% (*n* = 12/33) of the most affected genes were also found to be altered in squamous cell carcinoma (SCC) and melanoma (Table 2—genes highlighted in bold), strongly suggesting their involvement in the progression of IECs. Thus, these genes may represent promising targets for further investigation in therapeutic strategies to block or delay epidermoid malignant transformation.

Variants identified in the driver candidates were frequently located in specific DNA sites suggesting disease-related mutation hotspots. The hotspots were positioned in conserved protein regions, domains, and motifs (Supplementary Figure S1), strengthening a potential involvement in the disease. 

The functional protein association network analysis revealed three interaction clusters associated with the MHC protein complex, antigen processing, and presentation, suggesting again an altered mechanism to circumvent immune control (Figure 4B, Supplementary Table S9).

### 3.5. Recurrent Variants in NOTCH2 and USP8

After the investigation of the most altered genes, we conducted the analysis focusing on recurrent mutations observed in IECs. The most frequently observed alterations, affecting at least 50% (*n* = 3/6) of the cohort were identified in GSTT4 (p.S227P), KIR2DL1 (p.K237E), KIR2DL3 (p.E295D), MAGEC1 (p.T221S), OR9G1 (p.T83I), PABPC3 (p.H144R), PDPR (p.I106V), PRSS2 (p.R68C and p.C171Y), SLC35G4 (p.T263N), and USP8 (p.R657W). None of these variants has been previously associated with cancer (Figure 3C). 

Next, from all the recurrent variants identified, we investigated alterations that are likely to be deleterious, according to the Combined Annotation-Dependent Depletion (CADD) scores [108]. (Figure 4D, Supplementary Table S8). Recurrent and deleterious alterations included the two missense mutations p.R208W and p.R535W in GGT2, involving the gamma-glutamyl transpeptidase domain, p.S149N and p.V209M in HLA-DRB1, both positioned in the Ig-like C1-type domain and Beta-2 region, the missense p.Q677P in the EGF-like calcium-binding domain of NOTCH2, and p.P381S and p.G384C in UBXN11, both located adjacently to the UBX domain. In PDPR, the variants p.I106V and p.N562K are positioned in the chain and the GcvT domain of the protein, respectively. PDPR p.I106V, PABPC3 p.H144R, and USP8 p.R657W occurred in the highest frequency within the cohort, affecting 50% of patients. PDPR p.I106V is in the chain-pyruvate dehydrogenase phosphatase regulatory subunit, which is involved in the regulation of pyruvate metabolism. PABPC3 p.H144R is positioned in the RNA recognition motif (RRM) domain, which is the most prevalent RNA binding domain in eukaryotes, and it is responsible for the high-affinity binding to homopolymeric adenosines for the regulation of mRNA stability and translation initiation [109]. USP8 p.R657W is adjacent to the peptidase C19 domain of the deubiquitinase, which is important to recognize and hydrolyze the peptide bond at the C-terminal glycine of the ubiquitin (Figure 4E, Supplementary Figure S1). 

Among the recurrent and predicted deleterious variants, only the GGT2 p.R535W has been previously reported in cancer (COSM3766685) (Figure 4D). USP8 and NOTCH2 are well-known oncogenes that are frequently overexpressed in human cancers. The high expression of NOTCH2 and USP8 has been correlated to a worse prognosis in many tumor types [104,105,110,111], including SCC [84,110,111], and melanoma [85,105]. Consistently, USP8 and NOTCH2 mutations were characterized as gain-of-function alterations [112,113]. These observations suggest that the USP8 and NOTCH2 variants identified in IECs may enhance protein function and promote tumorigenesis. However, further experimental investigation is needed to fully characterize their roles in epidermoid formation and progression.

Considering NOTCH2 as a potentially significant driver of IECs, we noted that among the three clusters identified in the functional protein association network (Figure 4B), NOTCH2 indirectly interacts with clusters enriched by immune-associated genes. Notch signaling plays a crucial role in regulating various components of the tumor microenvironment, including immune and mesenchymal cells [114]. These observations suggest that an interplay between NOTCH signaling and immune cells within the IEC tumor microenvironment may influence immune-mediated oncogenic mechanisms.

### 3.6. Somatic Copy-Number Inference Strengthens the Involvement of Immune-Associated Genes in the Pathogenesis of IECs

The CNV analysis of each sample was performed by using CNVkit [33] (Supplementary Table S10). CNVs stratified by sample, CNV length, and hit counts by chromosome are shown in Supplementary Figure S2. The chromosome 19 was the most affected (Supplementary Figure S2). 

**Figure 5:**
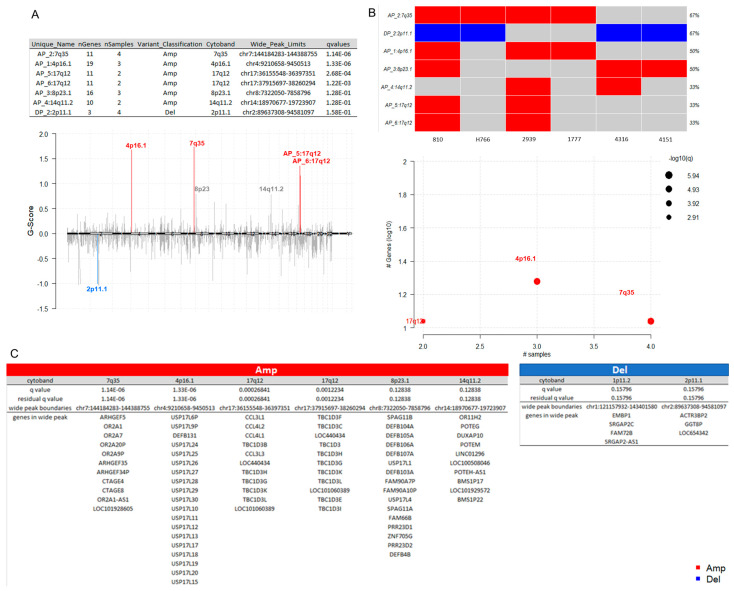
Somatic copy-number alterations in IECs. (**A**) Cytoband summary. The table at the top displays seven cytobands found to be altered in the cohort, including amplifications at 7q35, 4p16.1, 17q12 (AP_5 and AP_6), 8p23.1, and 14q11.2, as well as a deletion at 2p11.1. The chromPlot highlights the most significant events in the study (lower q values), specifically in cytobands 4p16.1, 7q35, 17q12, and 2p11.1. (**B**) Recurrently altered regions. The oncoplot and the bubble plot show the number of samples affected by frequently occurring copy-number events. Among them, the amplification of 7q35 (AP_5 and AP_6), 4p16.1, and 17q12 affected 67% (*n* = 4/6), 50% (*n* = 3/6), and 33% (*n* = 2/6) of the cohort, respectively (**C**) Genes located in wide peak regions. The wide peak boundaries illustrate the most likely gene targets within each aberrant cytoband region. Amplifications are indicated in red, while deletions are shown in blue. The red color indicates amplifications. The blue color indicates deletions. See also Supplementary Tables S10 and S11.

To identify somatic copy number regions that are significantly amplified or deleted across all six samples, the log_2_ ratios and segments generated with CNVkit [33] (Supplementary Table S10) were used as inputs for GISTIC [34] (Supplementary Table S11). The cohort analysis of the distributions of amplification and deletion events revealed seven cytobands significantly altered in the cohort, among them, amplifications of 7q35, 4p16.1, 17q12 (AP_5 and AP_6), 8p23.1, and 14q11.2, and the deletion of 2p11.1 (Figure 5A). Of these, the events with the lowest False Discovery Rate qvalues calculated for aberrant regions included the amplification of the cytobands 7q35 (AP_5 and AP_6), 4p16.1, and 17q12 (Figure 5A), affecting 67% (*n* = 4/6), 50% (*n* = 3/6), and 33% (*n* = 2/6) of the cohort, respectively (Figure 5B). The list of genes located in each wide peak region is shown in Figure 5C. Interestingly, the pattern of alterations in deubiquitinases and immune-related genes is also present at copy number levels. Recurrent amplifications were predominantly noted in DUB/ubiquitin-specific protease 17 (USP17) family, TBC1 domain protein family, and beta-defensins genes (DEFB4B, DEFB103A, DEFB104A, DEFB105A, DEFB106A, DEFB107A, and DEFB131) within the cohort, which overexpression plays a major role in the immune response to cancer [115,116,117,118,119,120].

### 3.7. Potential Targets in IECs 

To generate hypotheses about how the potential driver candidates of IECs might be targeted therapeutically or prioritized for drug development, we matched the list of the thirty-three gene driver candidates against the Drug-Gene Interaction database (DGIdb), a compendium of drug-gene interactions and potentially druggable genes, to prioritize drug-gene interactions [121,122]. The categorical analysis revealed the PTEN family which includes the TPTE gene. Variants in TPTE (p.L332P, p.R57Q, p.R84del, p.R84W) were observed in 70% of the cohort. Given that the pathway enrichment analysis indicated the significant enrichment by PI3K-Akt-mTOR and ECM pathways (Figure 3E,F), current PI3K, AKT, mTORC1/mTORC2, and PDK1 inhibitors may be interesting drug candidates for therapeutic intervention of IECs.

**Figure 6:**
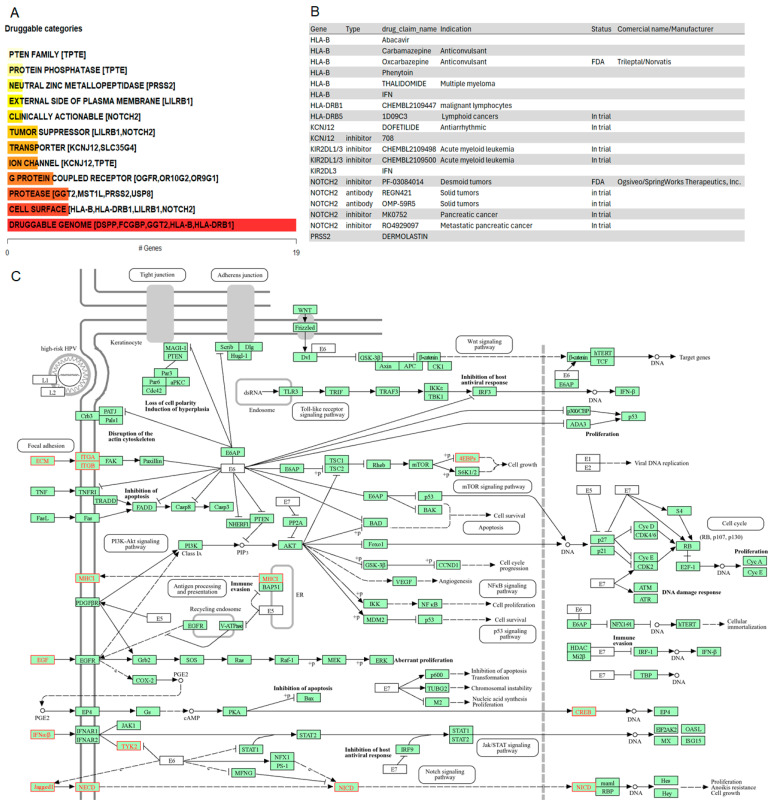
Drug-gene interactions and potentially druggable oncogenic mechanisms in IECs. (**A**) Druggable categories. The plot shows druggable gene categories along with up to top five genes involved in them. The categorical analysis revealed the PTEN family which includes the TPTE gene. NOTCH2 was identified as a clinically relevant target. (**B**) Drug-gene interactions. List of inhibitors and antibodies, including FDA-approved options to target the oncogenic drivers of IECs. (**C**) Crosstalk of potential mechanisms underlying the oncogenesis of IECs. Green boxes highlight the genes within the reference pathways, ensuring the pathway’s completeness. Red coloring in the pathway map indicates enriched disease-associated nodes. The figure highlights a crosstalk among focal adhesion, EGF, MHC, and antigen processing signaling cascades.

NOTCH2, whose genomic alterations p.Q677P, p.H1024Q, p.N2265K affected 50% of the IECs, was identified as a clinically relevant target for IECs (Figure 6A). The drug-gene interaction analysis indicated inhibitors and antibody candidates for targeted therapies (Figure 6B). Among these, the NOTCH2 inhibitor Nirogacestat (PF-03084014) is an FDA-approved chemotherapeutic agent marketed as Ogsiveo^®^ (SpringWorks Therapeutics). It is indicated for adult patients with progressing desmoid tumors requiring systemic treatment. Additionally, NOTCH2 inhibitors RO4929097 and MK0752 are currently in trial for pancreatic cancer. Furthermore, NOTCH2 antibodies OMP-59R5 (Tarextumab), and REGN-421 are under investigation for solid tumors (Figure 6B). 

To investigate additional oncogenic mechanisms driving intracranial epidermoid cysts that could be targeted by existing drugs, we examined the relationship between potential drivers of IECs and the top pathways identified in the enrichment analysis. The analysis demonstrated crosstalk among focal adhesion, EGF, MHC, and antigen processing signaling related to immune evasion and NOTCH2 signaling, indicated by the red coloring in the pathway. Together, these results provide evidence of multiple potential mechanisms underlying the oncogenesis of IECs (Figure 6C).

## 4. Discussion

There is an urgent need to understand the mechanisms and identify oncogenic drivers underlying the progression of IECs to improve treatment options. Due to the rarity of these tumors, limited research has been done; consequently, these patients continue to rely on surgery as the most effective treatment option, resulting in neurological impairment and poor outcomes. 

Next-generation sequencing has dramatically reshaped oncological treatment through the identification of genetic variants that provide prognostic information, and aid in therapeutic selection, resulting in hypothesis-driven clinical trials [21]. Since the genetics of IECs were completely unexplored, we sought to apply WES on resected IECs to improve our knowledge of the mechanisms of IEC’s oncogenic transformation and identify drivers of potential clinical value.

Tumor cells induce significant molecular, cellular, and physical changes in their surrounding tumor microenvironment (TME) to actively promote tumor development and progression [123]. The pathway enrichment analysis of the altered gene set identified in IECs revealed a strong association with the MHC complex. The pathway enrichment indicated involvement in antigen processing and presentation, ECM-receptor, focal adhesion, as well as PI3K-Akt-mTOR signaling. ECM components and the PI3K-Akt-mTOR signaling pathway play crucial roles in cell proliferation, adhesion, invasion, and migration [43,44]. Given that the TME comprises blood vessels, extracellular matrix, stromal and immune cells [123,124], our results indicate that genetic alterations in immune, ECM-receptor and adhesion genes may have a role in shaping the TME of intracranial epidermoid cysts.

IECs were characterized by an altered immune repertoire. The immune cells of the TME are the ones involved in the acquisition of immune escape mechanisms for tumor progression and development [123,124]. Tumor cells with stronger immunogenicity can be recognized and eliminated by the immune system, while some others can escape from it by several mechanisms and then develop into cancers [125]. Somatic mutations in immune genes affected 100% of the cohort, strongly indicating the involvement of a mechanism of immune evasion. Top altered immune-related genes included HLA and KIR genes. HLA class I-specific killer-cell immunoglobulin-like receptors (KIR) play a crucial role in regulating natural killer (NK) cell function in the elimination of malignancies. Alterations in the HLA expression patterns and/or changes in NK receptor modulation on effector cells can lead to an altered immune response, either enhancing or inhibiting antitumor cytolytic activity [125,126]. The relevance of KIR and HLA ligand gene background in the occurrence and outcome of certain tumors has been documented [127,128,129]. The high mutation burden observed in HLA and KIR genes suggests that these genes may be involved in mechanisms of immune evasion in IECs. Interestingly, Kulkarni et al. demonstrated that mice lacking the IL-1 receptor (IL-1R) (*IL1r^−/−^*) or deficient in IL1-β developed immunosuppression and multiple epidermal cysts after chronic UVB [24], suggesting that induced somatic events and an altered innate immune response may be involved in the initiation of epidermoid cysts, strongly corroborating our findings. 

To identify potential oncogenic drivers of IECs, we focused on the most altered genes identified in the cohort, resulting in a set of 33 driver candidates. Our literature review revealed that except PRB1, all other candidate drivers have previously been associated with cancer immune infiltration, TME, and/or endoplasmic reticulum (ER) stress, thereby supporting their role as oncogenic drivers in IECs.

Interestingly, many genes on the list of potential driver candidates have been implicated in SCC and melanoma. This may be related to the presence of epidermoid inclusions which consists of ectodermal and stratified squamous epithelial tissue embedded in connective tissue with keratin accumulation on the epithelium [12]. The transformation of IECs into malignant SCC [18] and melanoma [17] has been described. Targeting these overlapping genes could be a promising strategy to potentially block the malignant transformation of IECs.

Recurrent mutations in NOTCH2 and USP8 were identified in IECs, affecting 50% of the cohort. The most frequent alterations included USP8 p.R657W and NOTCH2 p.Q677P, observed in 50% and 30% of the cohort, respectively. Both USP8 and NOTCH2 are frequently overexpressed in human cancers [104,105,110,111]. Consistently, mutations on these genes confer upregulated protein functionality [112,113]. These observations suggest that the USP8 and NOTCH2 variants identified in IECs may enhance protein function, making them potential candidates for targeted therapy.

The missense p.Q677P in NOTCH2 is positioned in the EGF-like calcium-binding domain of the protein. The USP8 p.R657W is adjacent to the 14-3-3 binding motif (RSYSS). Somatic activating mutations in USP8 located between the amino acids 713 and 720 have been identified in (ACTH)-secreting neuroendocrine tumors. In ACTH pituitary adenomas, USP8 mutations are also positioned within or in close proximity to the 14-3-3 binding motif (RSYSS). Characterization of USP8 mutations in neuroendocrine tumors has shown that they promote oncogenic transformation through the activation of EGF receptor signaling [112]. Together, these observations suggest that alteration in USP8 and NOTCH2 may result in downstream dysregulation of EGF receptor signaling in IECs. Interestingly, aberrant interactions between EGF and ECM receptors result in the downstream activation of the PI3K-Akt-mTOR cascade [43]. Inhibition of USP8, NOTCH2, or EGFR deserved further investigation.

Many challenges are involved in drug development for IECs and other rare tumors, including a limited understanding of the tumor pathophysiology, molecular characteristics, and natural history. Additionally, there is a lack of timely access to molecular testing to determine eligibility for targeted therapies and difficulties enrolling sufficient patients for clinical trials. FDA-approved drugs for new indications may promote the development of safe and effective therapeutic approaches for these patients [22]. 

We conducted a drug-gene interaction analysis to generate hypotheses regarding how the potential driver candidates for IECs could be therapeutically targeted. The PTEN family category included the TPTE gene. Variants in TPTE were observed in 70% of the cohort. Given that the pathway enrichment analysis indicated significant involvement of PI3K-Akt-mTOR and ECM pathways, current inhibitors targeting PI3K, AKT, mTORC1/mTORC2, and PDK1 may be interesting drug candidates for therapeutic intervention of IECs.

NOTCH2 was highlighted as a clinically relevant target for IECs. Notch regulates both cancer cells and cancer stem cells as well as many components of the TME, including immune cells, fibroblasts, endothelial, and mesenchymal cells, cooperating with multiple signaling cascades to exercise its functions [114]. The analysis of the relationship between oncogenic drivers and altered signaling pathways in IECs provided evidence of crosstalk among focal adhesion, EGF, MHC, and antigen processing signaling where NOTCH signaling may contribute to tumor immune evasion. These observations highlight potential pathway nodes that could be targeted separately or in combination to tailor therapies for IECs. 

Finally, given the high genomic instability observed in deubiquitinases and the indication of tumor immune evasion mechanisms also observed at the copy-number level, proteasome inhibitors and immunotherapy may also represent interesting treatment options.

We acknowledge the limitations of our study. IECs are considered uncommon tumors, and our experimental design, which involved samples from a cohort of six patients, may be subject to bias due to the small sample size. We also acknowledge that combining newly diagnosed IECs with cases of recurrence (sample 4316) and progression (sample H766) might induce a bias in the results. These limitations also complicate the correlation of molecular findings with clinical data, making it challenging to identify potential biomarker candidates. Furthermore, experimental validation is difficult due to the lack of suitable in vitro or in vivo models. Despite these challenges, molecular information on IECs remains scarce, and the relationship between immune system modulation and tumorigenesis in IECs has not been well characterized. The genetic data presented here highlight potential driver candidates that could be targeted with existing drugs and contribute to a better understanding of the molecular mechanisms underlying IEC oncogenesis. Further investigation is needed to elucidate how these mutations may induce changes in the surrounding non-tumor immune cells of the TME, contributing to tumor immune escape mechanisms. However, the highly reproducible somatic patterns of an altered immune repertoire, along with the crosstalk between PI3K-Akt-mTOR and ECM signaling, provide strong evidence of an immune escape mechanism in IECs, in which NOTCH2 may play a role. This mechanism may be influenced by similar underlying molecular factors across most patients, highlighting new opportunities to reverse tumor immune escape and progression. Additionally, variations in NOTCH2 and USP8 could pave the way for novel investigations and the development of tailored approaches to treat IECs.

## 5. Conclusions

We investigated the somatic landscape of IECs to gain insights into tumor biology and identify potential drivers for targeted therapies. While surgery remains the most effective treatment for these patients, our findings highlight key altered genes associated with the immune system and TME, suggesting mechanisms of immune evasion. Notably, frequent amplifications in deubiquitinases and beta-defensins further emphasize the role of immune mechanisms in oncogenic transformation. We also identified recurrent and deleterious alterations in NOTCH2 and USP8, which may be clinically relevant drivers of IECs. Finally, we provide evidence that the crosstalk between PI3K-Akt-mTOR and ECM signaling may have an important function in modulating the immune escape mechanism in IECs, in which NOTCH2 may play a crucial role.

## Figures and Tables

**Table 1:** Demographics and Tumor Characteristics.

Sample	Age	Sex	Symptoms	Tumor Location	Previous Treatment	Extent of Resection	Recurrence Progression (Months)
H766	41	F	Syncope Headache Vision	Suprasellar	None	STR	7
4151	57	F	Neuralgia	Cerebellopontine	None	GTR	None
1777	30	M	Epilepsy	Cerebellopontine	None	GTR	None
2939	34	F	Vision Adrenal insufficiency	Suprasellar	None	STR	None
810	27	F	Headaches Emesis Hydrocephalus Ataxia Nerve palsy Cognitive	Cerebellopontine	None	nGTR	None
4316	32	F	Headache	Middle fossa	2	GTR	60

GTR = Gross total resection; STR = Subtotal resection; nGTR = near Gross Total Resection; F: Female; M: Male. See also Supplementary Table S1.

**Table 2:** Characteristics of potential drivers of IECs.

Hugo Symbol	Status	Cancer Type	Oncogenic Function	References
**DSPP**	**Upregulation**	**Head** **Neck** **SCC**	**ER stress invasion** **Metastasis** **Angiogenesis** **EMT**	[45,46]
**FCGBP**	**Aberrant regulation**	**Glioma** **Colorectal** **SCC**	**Immune infiltration**	[47,48,49]
FMN2	NR	Osteosarcoma	Regulation of tumor suppressor pathway mediated by p14ARF	[50]
**FRAS1**	**Upregulation**	**SCC**	**Cell growth** **Metastasis**	[51]
**GGT2**	**Upregulation**	**Endometrial Gastrointestinal** **SCC** **Melanoma**	**Metabolic signaling** **Cell growth**	[52,53,54]
**GSTT4**	**Mutation**	**Acute leukemia** **SCC**	**Cellular detoxification**	[55,56,57,58]
**HLA-B**	**Mutation** **Downregulation**	**Breast** **Colon** **Rectal** **SCC** **Melanoma**	**Immune infiltration**	[59,60,61,62]
**HLA-DRB1**	**Upregulation** **Fucosylation**	**Melanoma** **Lung** **SCC**	**Immune infiltration**	[63,64,65,66,67]
**HLA-DRB5**	**Upregulation**	**Melanoma** **Lung** **SCC**	**Immune infiltration**	[65,66,67]
IGFN1	Aberrant expression Mutation	NSCL Renal	Metastasis	[68,69]
**KCNJ12**	**Mutation**	**Glioblastoma** **Prostate** **SCC**	**Ion channel**	[70,71,72]
**KIR2DL1**	**Mutation**	**Glioblastoma** **Melanoma**	**Immune infiltration**	[73,74]
**KIR2DL3**	**Mutation**	**Glioblastoma** **Melanoma**	**Immune infiltration**	[73,74]
**KIR2DS1**	**Mutation**	**Glioblastoma** **Melanoma**	**Immune infiltration**	[73,74]
**KIR2DS2**	**Mutation**	**Glioblastoma** **Melanoma**	**Immune infiltration**	[73,74]
**KIR3DL1**	**Mutation**	**Glioblastoma** **Melanoma**	**Immune infiltration**	[73,74]
**LILRB1**	**Upregulation**	**Melanoma** **Multiple tumors**	**Immune infiltration**	[75,76,77]
MAGEC1	Aberrant expression	Cholangiocarcinoma Hepatocellular	Immune infiltration Apoptosis Cell cycle	[78,79]
MST1L	Downregulation	Breast	Cell differentiation Adhesion Migration Apoptosis	[80]
**NOTCH2**	**Aberrant expression** **Mutation**	**SCC** **Melanoma** **Multiple tumors**	**Immune infiltration** **Stem-like proliferation**	[81,82,83,84,85]
**OGFR**	**Aberrant expression** **Mutation**	**Gynecological** **Ovarian** **SCC**	**Cell proliferation** **Cell cycle**	[86,87,88]
OR10G2	Aberrant expression	Multiple tumors	Cell differentiation Invasion Metastasis	[89,90]
OR9G1	Aberrant expression	Multiple tumors	Cell differentiation Invasion Metastasis	[89,90]
PABPC3	Aberrant expression	Pancreas Osteosarcoma	Immune infiltration Cell proliferation Metastasis	[91,92]
PDPR	Downregulation	Thyroid	NR	[93]
PRB1	NR	NR	NR	NR
PRSS2	Upregulation	Gastric	EMT Metastasis Microenvironment	[94,95]
RBMX	Downregulation	Multiple tumors	Tumor development	[96,97]
SLC35G4	Upregulation	Glioma Breast	Immune infiltration	[98,99]
TPTE	Upregulation	Lung Prostate	Immune infiltration	[100,101]
UBXN11	Upregulation Mutation	Multiple tumors	Tumor progression	[102]
**USP8**	**Upregulation** **Mutation**	**SCC** **Melanoma** **Multiple tumors**	**Immune infiltration** **Tumor progression** **Drug resistance**	[103,104,105]
ZNF717	Mutation	Colorectal Hepatocellular	NR	[106,107]

Gene alterations previously reported for Squamous Cell Carcinoma and or melanoma are highlighted in bold letters. SCC: Squamous Cell Carcinoma; NSCL: Non-small-cell lung cancer; EMT: Epithelial-Mesenchymal Transition; ER: Endoplasmic Reticulum; NR: Not Reported.

## Data Availability

The original data presented in the study are openly available in the NCBI’s Sequence Read Archive (SRA).

## Supplementary Materials

The following supporting information can be downloaded at: https://www.mdpi.com/article/10.3390/cancers16203487/s1. Figure S1: Mutation hotspots in IECs; Figure S2: Summary of somatic copy-number alterations in IECs; Table S1: Clinical features; Table S2: Software and databases; Table S3: IEC variants; Table S4: GO analysis; Table S5: KEGG pathway enrichment; Table S6: WikiPathway enrichment; Table S7: Reactome pathway enrichment; Table S8: Driver Candidates; Table S9: Protein-protein Interaction Networks; Table S10: Copy Number Variation with CNVKit; Table S11: GISTIC Summaries.
