# Supplementary material for: Whole Exome Sequencing of Intracranial Epidermoid Cysts Reveals Immune-Associated Mechanistic and Potential Targets
Source: Cancers (Basel). 2024 Oct 15;16(20):3487. doi: 10.3390/cancers16203487 (PMC11506683; doi:10.3390/cancers16203487)
Supplement: Supplementary file 1 [file cancers-16-03487-s001.zip › Figures S1 and S2.pdf]

cancers

Article

Somatic landscape of intracranial epidermoid cysts reveals immune-associated mechanistic and potential actionable targets.

Shruthi Kondaboina[1], Oscar Parrish[1], Carolina Parada[1]*, Manuel Ferreira Jr[1]*

[1] University of Washington Medical Center, Department of Neurological Surgery 1;
SK: shruthi-kondaboina@uiowa.edue-mail@e-mail.com
OP: oscarparrish3@gmail.com
CP: cdsp@uw.edu
FJ: manuelf3@uw.edu*
Correspondence: CP: cdsp@uw.edu; MFJ: manuelf3@uw.edu

Supplementary Figures

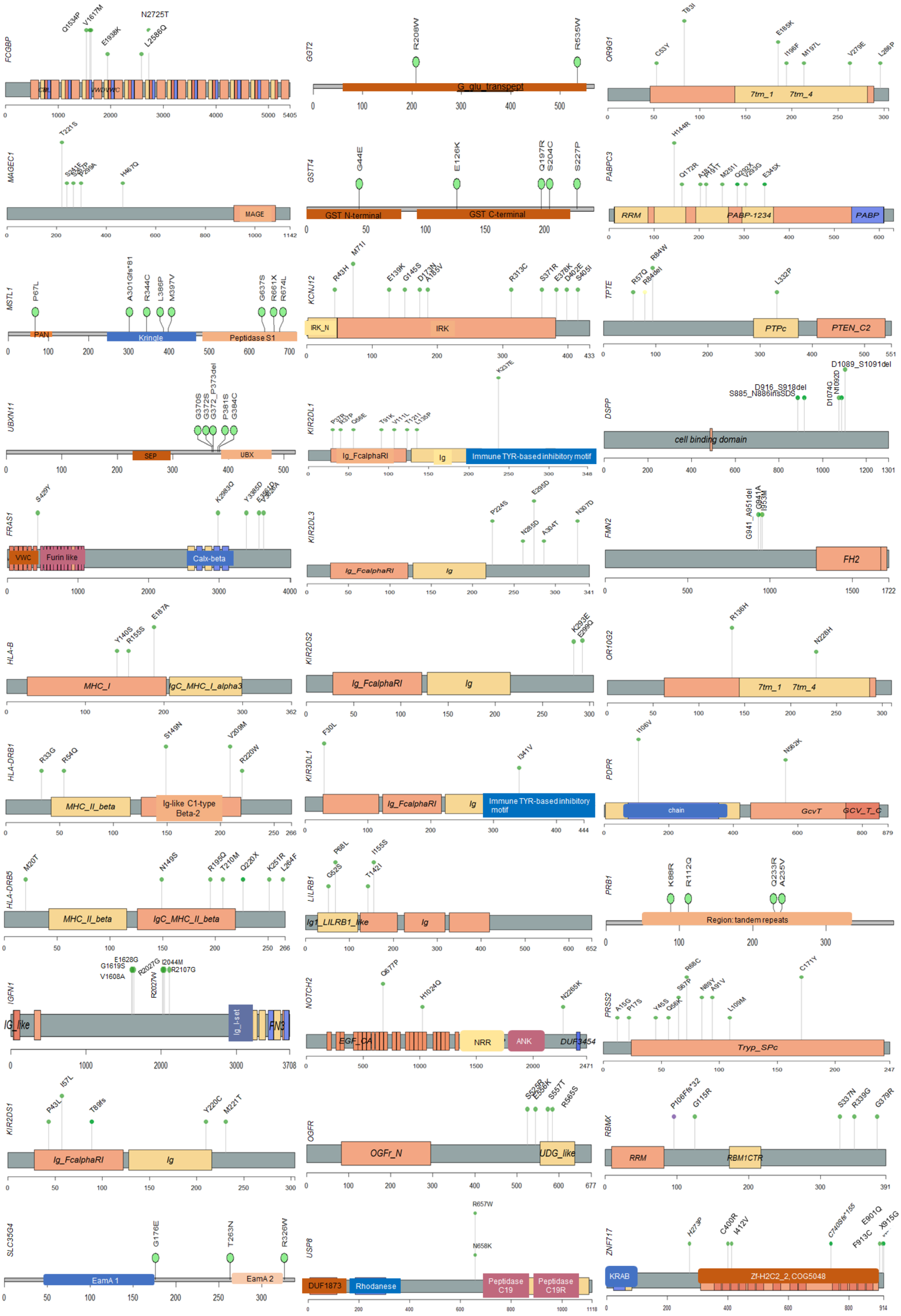
FCGBP
Q1534P
V1617M
E1938K
N2725T
L2586Q
VWC
WDWC
GGT2
R208W
R535W
G_glu_transpept
OR9G1
T83I
C53Y
E185K
I196F
M197L
V279E
L286P
7tm_1
7tm_4
MAGEC1
T221S
H467Q
MAGE
GSTT4
G44E
E126K
Q197R
S204C
S227P
GST N-terminal
GST C-terminal
PABPC3
H144R
Q172R
E345X
RRM
PABP-1234
PABP
MSTL1
P67L
A301Gfs*81
R344C
L386P
M397V
G637S
R661X
R674L
PAN
Kringle
Peptidase S1
KCNJ12
R43H
M71I
E139K
G145S
R313C
S371R
IRK_N
IRK
TPTE
R57Q
R84W
R84del
L332P
PTPc
PTEN_C2
UBXN11
G370S
G372S
G372_P373del
P381S
G384C
SEP
UBX
KIR2DL1
K237E
Ig_FcalphaRI
Ig
Immune TYR-based inhibitory motif
DSPP
D1089_S1091del
D916_S918del
S885_N886insSDS
D1074G
N1092D
cell binding domain
FRAS1
S429Y
K2983Q
Y3385D
VWC
Furin like
Calx-beta
KIR2DL3
E295D
P224S
N285D
A304T
N307D
Ig_FcalphaRI
Ig
FMN2
G941_A951del
G941A
I953M
FH2
HLA-B
Y140S
R155S
E187A
MHC_I
IgC_MHC_I_alpha3
KIR2DS2
Ig_FcalphaRI
Ig
OR10G2
R136H
N228H
7tm_1
7tm_4
HLA-DRB1
R33G
R54Q
S149N
V209M
R220W
MHC_II_beta
Ig-like C1-type Beta-2
KIR3DL1
F30L
I341V
Ig_FcalphaRI
Ig
Immune TYR-based inhibitory motif
PDPR
I106V
N562K
chain
GcvT
GCV_T_C
HLA-DRB5
M20T
N149S
R195Q
T210M
Q220X
K251R
L264F
MHC_II_beta
IgC_MHC_II_beta
LILRB1
P68L
I155S
G52S
T142I
Ig1_LILRB1_like
Ig
PRB1
K88R
R112Q
Q233R
A235V
Region: tandem repeats
IGFN1
E1628G
G1619S
V1608A
R2027G
R2027W
I2044M
R2107G
IG_like
Ig_I-set
FN3
NOTCH2
Q677P
H1024Q
N2265K
EGF_CA
NRR
ANK
DUF3454
PRSS2
C171Y
R68C
A15G
P17S
Y45S
Q56K
N86Y
A91V
L109M
Tryp_SPc
KIR2DS1
I57L
P43L
T89fs
Y220C
M227T
Ig_FcalphaRI
Ig
OGFR
S552R
S557T
R565S
OGFr_N
UDG_like
RBMX
P106Ffs*32
G115R
S337N
R339G
G379R
RRM
RBM1CTR
SLC35G4
G176E
T263N
R326W
EamA 1
EamA 2
USP8
R657W
N658K
DUF1873
Rhodanese
Peptidase C19
Peptidase C19R
ZNF717
H273P
C400R
I412V
C740Sfs*155
E901Q
F913C
X915G
KRAB
Zf-H2C2_2, COG5048

Figure S1: Mutation hotspots in IECs. Lollipop plots of mutation distribution in the 34 top altered genes. Variations were frequently located on specific DNA sites suggesting disease-related mutation hotspots. The hotspots were often positioned on conserved protein regions, domains and motifs, strengthen their role as oncogenic drivers of IECs.

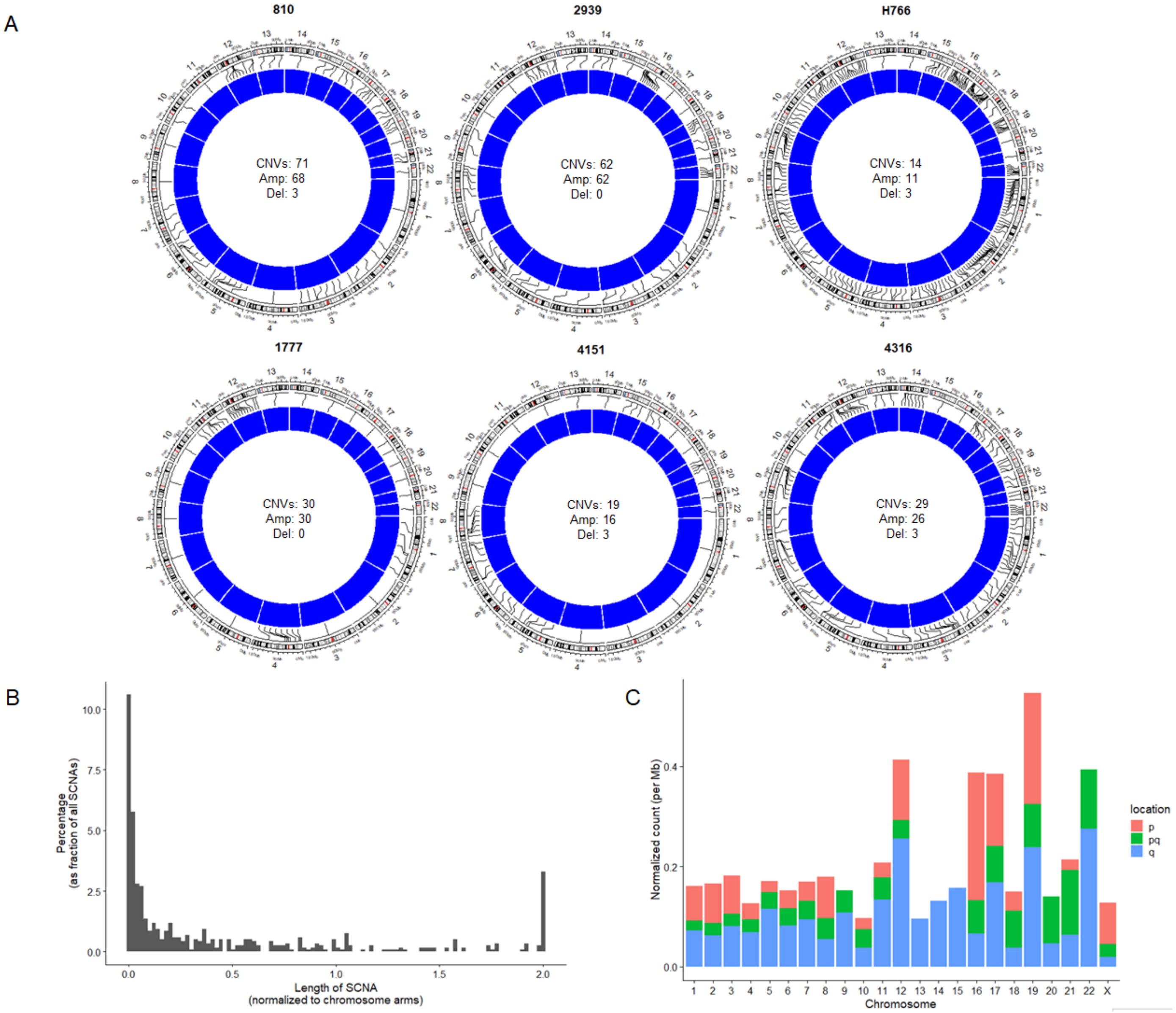

Figure S2: Summary of somatic copy-number alterations in IECs. (A) Circos plot of the copy number variation frequency profile in each sample. (**B)** Histogram of copy-number distribution by segment length of SCNAs and percentage (as fraction of all SCNAs). (**C)** Stacked bar chart of copy-number distribution by chromosome. See also Supplementary Table S10 and S11.
